# Minimally Invasive Spinal Interventions for Back and Neck Pain in the Pediatric Population: a Systematic Review

**DOI:** 10.1007/s11916-026-01521-4

**Published:** 2026-07-16

**Authors:** David Jevotovsky, Ruben Dovlatyan, Sara Mermelstein, Dylan Banks, Roi Medina, Austin Dukat, Christopher Lee, Salman Hirani, Salvador Portugal, Tracy Espiritu McKay

**Affiliations:** 1 New York University Langone Medical Center, New York, NY USA; 2 Rush University Medical Center, Chicago, IL USA; 3 University of California Davis Medical Center, Sacramento, CA USA; 4 Oregon Health & Science University, Portland, OR USA

## Abstract

**Objective:**

To evaluate the effectiveness and safety of spinal interventions for pediatric patients with chronic back and neck pain who failed conservative management.

**Design:**

Systematic review.

**Methods:**

Following PRISMA 2020 guidelines, a comprehensive search of PubMed, Embase, Web of Science, and Cochrane Central was performed through August 2024. Eligible studies included clinical reports of interventional procedures for pediatric spine related pain. Primary outcomes were pain reduction measured by subjective scales or validated instruments; secondary outcomes included safety and adverse events. Risk of bias and certainty of evidence were assessed using the MASTER and GRADE scales.

**Results:**

Of 578 identified studies, 8 met inclusion criteria, encompassing 258 pediatric patients. Interventions included epidural steroid injections (*n* = 227), sacroiliac joint injections (*n* = 13), facet injections (*n* = 2), ozone discolysis (*n* = 2), medial branch blocks (*n* = 1), interspinous ligament injections (*n* = 1), and microdiscectomy (*n* = 1). Across all studies, some degree of pain relief was reported, with functional improvement noted when measured. Adverse events were rare, mild, and transient, including a post-procedural headache and a transient sciatic motor block. No major long-term complications were reported. Evidence certainty was graded as ‘very low’ due to small sample sizes, observational designs, and heterogeneous methodologies.

**Conclusions:**

Preliminary evidence suggests spinal interventions may provide pain relief and functional improvement for carefully selected pediatric patients refractory to conservative measures, with a safety profile comparable to adults. However, evidence remains limited and heterogeneous. Larger, controlled studies are needed to guide clinical practice and inform future guidelines.

**Supplementary Information:**

The online version contains supplementary material available at 10.1007/s11916-026-01521-4.

## Introduction

Back and neck pain in the pediatric population have a negative impact on both physical and emotional well-being. The prevalence of chronic pediatric low back pain varies, with reported rates ranging from approximately 6–19% [1, 2], while neck pain prevalence estimates range from 17–35% [1, 3, 4]. Recent studies indicate a sustained upward trend in both conditions, as well as increasingly higher rates among older adolescents [2, 3, 5]. Factors including female sex, excess weight, and lower socioeconomic status have also been associated with a greater likelihood of experiencing chronic low back pain in child and adolescent populations [2]. Additionally, year-long and early sport specialization puts younger athletes at a greater risk for low back pain [6]. Nonspecific pain (e.g. nonspecific lower back pain) is the most common source of axial pain in pediatric populations while pathologic diagnoses, such as fractures or tissue specific injuries such as myalgias, are less common [7]. Given the growing prevalence of back and neck pain in the pediatric population, along with evidence indicating that pain during childhood increases the likelihood of pain persisting into adulthood [8], there is a need for both conservative and interventional strategies to mitigate the progression to chronic pain later in life.

Similar to adult populations, the management of pediatric back and neck pain generally begins with conservative treatment strategies including activity modification, multidisciplinary therapy and rehabilitation modalities, and oral pharmacologic agents. While adults who fail conservative management may receive epidural steroid injections and radiofrequency ablations, there is greater hesitation to use steroids in the pediatric population due to concerns about adverse effects in skeletally immature individuals [9, 10]. Endogenous cortisol suppression, growth hormone suppression, and compromised bone health have all been described [9, 10]. Spinal interventions in the pediatric population also present unique technical challenges due to the anatomic differences in the developing spine, as well as the high prevalence of structural deformities that may complicate procedural access and imaging [11]. Regardless, existing literature suggests there may be a role in select pediatric patients who failed conservative measures.

This review aims to summarize the published literature and evaluate the level of evidence of spine interventions for pediatric back and neck pain. To our knowledge, this is the first systematic review dedicated to this topic. Given the limited literature and lack of consensus on the management of pediatric spine pain, this review seeks to inform clinical decision-making while identifying gaps that warrant further investigation.

## Methods

### Overview

The Preferred Reporting Items for Systematic Reviews and Meta-analysis (PRISMA) 2020 guidelines were followed in the formulation of this review [12]. No a priori protocol was published in a peer-reviewed journal. The review was registered with the International Prospective Register of Systematic Reviews (PROSPERO) and approved in August 2024 (ID: CRD42024554256).

### Study Eligibility

The present study included clinical studies on the use of spinal interventions in pediatrics patients for the initial abstract review. Interventions included but were not limited to epidural steroid injections, interventions to the facet joints or medial branches, sacroiliac joint injections, and interventions to the discs. The primary outcome of interest was the effectiveness of spine interventions in alleviating pain, assessed via the Numerical Pain Rating Scale (NPRS), or other pain-related outcome scores, including subjective relief. As a secondary outcome, this review examined safety of spinal interventions in the pediatric population, with a focus on any reported adverse events (AE). Only studies published in English or available in English translation were included in this review. Exclusion criteria included non-human subjects and if the study only described adult participants older than 18 years of age. Reviews, letters to the editor, and conference abstracts were also excluded.

### Search Strategy

A comprehensive literature search was conducted by an institutional librarian (RM, Acknowledgements) in August 2024, to identify all relevant articles on spine interventions for the treatment of pediatric pain. A comprehensive review of PubMed, Embase, Web of Science and Cochrane Central Register of Controlled Trials were searched using terms such as "back pain", "neck pain", “epidural”, “facet” and “pediatric” from database inception to August 2024. The complete search strategy is outlined in supplemental data file 1. Computer deduplication of resulting studies followed.

### Study Selection

Two independent reviewers (RD and SM) independently assessed the studies for inclusion based on their relevance to spine interventions’ effectiveness in the pediatric pain population. Discrepancies were resolved after discussion with a third reviewer (DSJ). Full text evaluation of relevant studies followed abstract screening, considering the aforementioned inclusion criteria. Outcomes extracted included the Visual Analogue Scale (VAS), other pain-related outcomes, and safety measured by AE.

### Data Extraction and Assessment of Study Quality

Data collection from full-text articles was performed independently by three reviewers (SM, RD, AD) using a standardized data extraction sheet, with a fourth investigator (DSJ) available to to resolve any discrepancies. Extracted variables included patient demographics, study design features, procedural details, outcomes, and adverse events. The primary outcome was change in reported pain levels. Study quality was assessed in accordance with the American Medical Association's Journal of Ethics’ guidelines for Rating Evidence in Medical Literature [13]. The Methodological Standards for Epidemiological Research Scale was utilized to assess internal validity and identify potential sources of bias (supplemental file 2) [14]. Strength of the overall body of evidence was determined using The Grading of Recommendations Assessment, Development and Evaluation Scale [15].

### Analysis and Post hoc Changes to a Priori Protocol

Heterogeneity across study outcome measures and follow-up durations made a meta-analysis inappropriate for summarizing the evidence. Therefore, we present a systematic review, presenting our findings to consolidate the existing evidence and identify key trends and outcomes.

## Results

### Literature Search

**Fig. 1:**
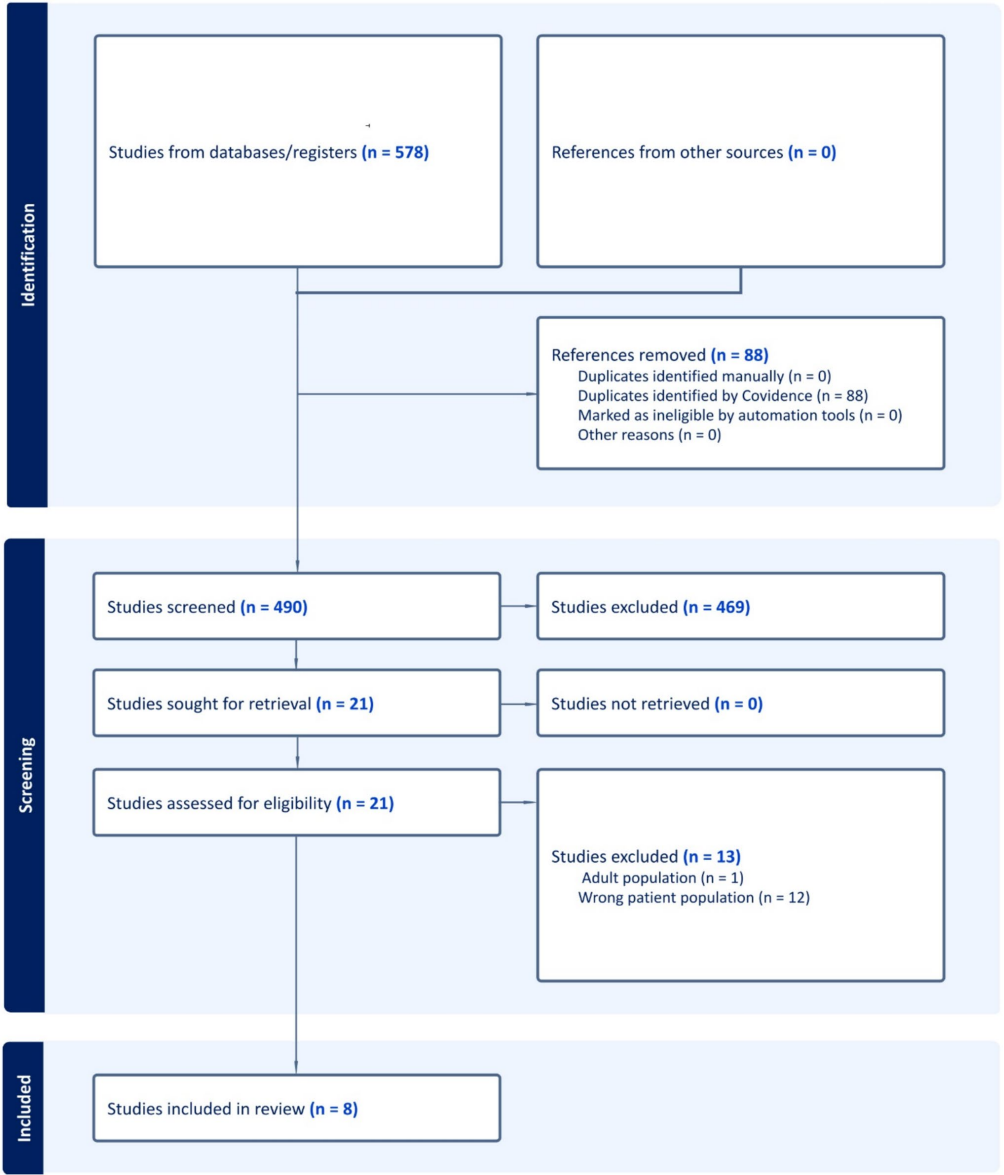
PRISMA flow diagram

A total of 578 studies were identified through the initial database search. After removal of duplicates, 490 titles and abstracts were screened before 21 studies underwent full-text review. Of these, 8 studies met all inclusion criteria and were included in the final analysis (Fig. 1, PRISMA flow diagram). Reason for exclusion during full-text review can be found in supplemental file 3.

### Study Characteristics

The 8 included studies collectively represented 258 pediatric patients undergoing minimally invasive spine interventions. Sample sizes ranged from single case reports to cohorts of over 100 patients. Of note, 224 of the included patients came from a single retrospective cohort study on radiculopathy with or without back pain treated with epidural steroid injection (ESI) [16]. The mean age of participants varied across studies, typically ranging from 12 to 18 years, with most patients being adolescents. Both sexes were represented.

The interventions examined included ESIs (*n* = 227), sacroiliac joint injections (*n* = 13), facet joint injections (*n* = 2), ozone discolysis (*n* = 2). medial branch blocks (*n* = 1), tubular microdiscectomy (*n* = 1), and interspinous ligament injections (*n* = 1). The most frequent pain etiologies reported were nonspecific low back pain, herniated discs with or without radiculopathy and sacroiliac joint pain. There were also cases of tethered cord syndrome, Baastrup’s disease, and sacral facet fractures. Follow-up duration ranged from 5 days post-procedure to 4 years, with 5 studies providing outcomes at greater than or equal 6 months of follow-up. Two studies did not provide specifics on follow-up time points [17, 18]. Excluding these two studies, the median patient follow-up time was 6 months in the present review.

### Outcome Assessments

Across studies, reduction in pain intensity, otherwise referred to as pain relief, was the most consistently reported outcome. Pain was most frequently measured using a subjective pain relief scale, though studies also utilized the VAS, with most patients demonstrating improvement following intervention. Studies compared baseline preintervention pain scores to postintervention pain scores at follow up specified by the individual studies. Also of note, 2 studies commented on return to sport in pediatric athletes who had undergone spinal interventions.

All 8 studies reported some degree of pain improvement following spinal interventions, and when assessed, a return to sport amongst pediatric patients (Table 1) [16–23]. Although most studies included small sample sizes of fewer than 14 patients, one large retrospective cohort study by Kurgansky et al. was identified, which followed 224 patients treated with ESIs. Among these, 174 patients (76.0%) had lumbar disc herniation with radiculopathy, and median subjective pain relief scores improved significantly at all follow-up points (median follow-up 799 days). In a subgroup of 61 patients who completed prospective follow-up questionnaires, subjective pain relief remained favorable, and improvements were also seen in functional and quality-of-life measures, including the Oswestry Disability Index (ODI), Functional Disability Inventory (FDI) and Pediatric Quality of Life Inventory (PedsQL).

**Table 1:** Characteristics of the included clinical studies

#	Title	First author (Year)	Study design	Level of evidence	Patient population (mean age, % female)	Treatment group	Prior treatment modalities	Pain outcome assessed	Follow-up length of time	Analgesic outcomes	Adverse effects	Funding
2	Pediatrics severe low back pain by disc herniation: an uncommon entity	Tiendrebeogo/Zabsonre (2024)	Case series	4	Three patients with severe LBP secondary to disc herniation (15 years, 33% female)	ESI (dosing, medication and level unavailable) and ozone L5-S1 Discolysis Ozone L5-S1 Discolysis ESI (dosing, medication and level unavailable) after parenteral tramadol, thiocolchicoside and methyl prednisolone	Acute hospitalization with IV paracetamol, morphine, thiocholchicside nad methyl prenisolone Acute hospitalization with parenteral paracetamol + tramadol, a muscle relaxant and methyl prednisolone "The usual analgesics" and acute hospitalization with parenteral paracetamol, tramadol, thiocolchicoside and methyl prednisolone	VAS	3 months 3 months 5 days	(1) VAS from baseline 10/10 to 1/10 post-procedure At f/u, "the evolution was good without any treatment." (2) VAS from baseline 9/10 to 0/10 post-procedure "The course was favorable…without [additional] treatment." (3) VAS Baseline 9/10 to 0/10 at 5 days post-treatment	None documented through entire series	none
58	Sacroiliac joint pain in adolescents: Diagnostic and treatment challenges	Cucchiaro (2022)	Case series	4	Thirteen patients with SI joint pain (18 years, 7.7%)	Methylprednisolone 20 mg in 2–3 cc of 0.5% bupivicaine, injected into SIJ 1 patient with unilateral injection, required repeat injection at 2 month f/u 2 patients with bilateral injections, required repeat injection at 2 month f/u 10 patients wtih single bilateral injection at one time point only	NSAIDs in combination with gabapentin and/or cyclobenzaprine and antidepressants	Subjective reports on pain improvement	6 months	6 patients (46%): immediate improvement in pain 4 patients (31%): complete pain at 6-month follow-up 1 patient (8%) with unilateral pain resolution following bilateral SIJ injection 6 patients (46%): no benefit 3 patients (23%): pain initial improvement but pai flair about 2-months after procedure. (all repeated injection, 2 of which had long-lasting relief, 1 whose pain recurred 12-months later)	1 patient had experienced sciatic nerve block with partial motor block within few minutes of injection. Spontaneous resolution of motor block within 24 h, and of numbness within 48 h	Not listed
60	Baastrup’s Disease in Pediatric Gymnasts	Ali (2022)	Case series	4	Two female pediatric athletes with Baastrup’s disease (17 years, 100%)	Bilateral T11, T12, and L1 medial branch blocks using 6 mL of 0.2% ropivacaine mixed with 60 mg Depo-Medrol Three separate interspinous ligament injections between L2 and L3 over four years, each with 1 mL of 0.2% ropivacaine and 40 mg Kenalog	Relative rest, PT, massage Relative rest, PT	Subjective pain relief and return to activity; no numeric scale used	1 month month, 1 year, 2 years, 4 years	1 month: complete pain relief, return to full gymnastics activity without recurrence Sustained pain relief after each injection with progressive return to activity Improvement at 1-year and again at 4-year follow-up. Pain recurrance between follow-ups was persistently less severe than initial presentation, and may have been caused by ongoing physical activity between the follow-up phases	None Reported	none
113	Low back pain in adolescent athletes: Comparison of diagnoses made by general orthopedic surgeons and spine surgeons	Yamashita, Kazuta (2019)	Retrospective case study	4	Epidemiologic study of 69 adolescent athletes with nonspecific back pain (15.2 ± 2.3 years years, 21.7%) *Case studies within:* 1) 15-year-old male with nonspecific LBP 2) 18-year-old male with discogenic pain	*Case studies within:* 1) Facet block injection 2) Discography + nerve block injection	None reported	Subjective pain relief	Not documented	Complete pain resolution	none documented for either case study	none
153	Tubular approach to minimally invasive microdiscectomy for pediatric lumbar disc herniation	Montejo, Julio (2018)	Retrospective cohort study	2B	Twelve patients with lumbar disc herniation (17 ± 1.6 years, 58%)	Microdiscectomy patients (50% of which received ESI prior to surgery)	NSAIDs, opioids, PT, chiropractic thearpy, ESI	ODI Subjective relief	Median follow-up: 2.8 years	ODI: low-back pain score of 19.7% (SEM 2.8%) at the last follow-up Subjective relief: Symptoms completely resolved or significantly improved in 91%	No operative or post operative adverse effects 30 days post-op One patient experienced a recurrence of radicular symptoms at 18 months post-procedure and required a second same-level MIS tubular microdiscectomy, which successfully resolved the patient’s symptoms	none
184	Spinal cord stimulation for recurrent tethered cord syndrome in a pediatric patient: Case report	Tyagi, Rachana (2018)	Case study	4	12 year-old female with recurrent tethered cord syndrome (12 years, 100%)	Spinal cord stimulation with leads at T8 Caudal ESI (dosing and medication unavailable)	NSAIDs, gabapentin, duloxetine, oxycodone/acetaminophen, PT, TENS	VAS	Not documented after ESI 6 weeks, 6 months, 10 months	"Minimal relief" from epidural injection Baseline: 8/10 6 weeks: 1/10 6 months: 1–2/10 10 months: 4/10	none documented	none
193	Epidural Steroid Injections for Radiculopathy and/or Back Pain in Children and Adolescents < i > A Retrospective Cohort Study With a Prospective Follow	Kurgansky, Katherine (2016)	Retrospetive cohort study	2B	224 patients with radiculopathy and/or back pain (range: 9 to 20 years, 62.5% female)	ESI - Approach: ILESTI > TFESI > Caudal - Medication: Triamcinolone > Depo-Medrol > Triamcinolone + Depo-Medrol > Dexamethasone	Back surgery before their first ESI (*n* = 20): discectomy, laminectomy, or foraminotomy (*n* = 9) spinal fusion and instrumentation (*n* = 10) spinal fusion and discectomy (*n* = 1)	Subjective pain relief	Median follow-up: 799 days	Among patients who had a lumbar herniation and radiculopathy, overall pain scores were low	No infection, allergic reaction, visualized dural puncture,or systemic steroid-related complication was identified One patient without a recognized dural puncture had a postural headache that resolved after an epidural blood patch 4 days later	none
263	Sacral Facet Fractures in Elite Athletes	Skaggs, David L (2012)	Case Study	4	14 year-old female with facet fractures (14 years, 100% female)	Steroid injections into the L5 right SAP fracture and then another into the left SAP fracture two days later (dosing and medication unavailable)	Relative rest	self-reported pain relief	Not documented	After L5 injection, patient reported "limited pain relief" After S1 injection, patient reported "virtually total pain relief"	It was decided to remove the S1 fracture fragment surgically	none

Most studies reported follow-up between 3–10 months. Three provided longer-term data of 2 to 4 years) Kurgansky et al. reported a median follow-up of 799 days, with over half of patients (51.5%) followed for ≥ 2 years. Montejo et al. reported a median follow-up of 2.8 years, while Ali et al. demonstrated sustained improvement at 4 years.

Functional outcomes were assessed less consistently. When measured, instruments such as the ODI suggested parallel improvements in function alongside pain relief. For example, Montejo et al. reported an ODI of 19.7% (SEM 2.8%) at post-procedure follow-up. Patient-reported satisfaction was subjectively high in case series where it was assessed, though validated quality-of-life instruments were rarely employed.

### Adverse Events

Adverse events were infrequent, mild and transient. One case of transient partial sciatic motor block following a sacroiliac joint injection was reported, which resolved spontaneously within 24 h. The patient’s associated numbness resolved within 48 h [20]. An additional case of postural headache after an epidural injection, attributed to an unrecognized dural puncture, which resolved with an epidural blood patch on postoperative-day four [16].

No long-term procedural-related complications or serious adverse events were reported. One patient experienced a recurrence of radicular symptoms 18-months after microdiscectomy which resolved after a second same level minimally invasive tubular microdiscectomy [21].

### Quality of Evidence

All included studies were observational in design; no randomized controlled trials were identified. Common limitations included small sample sizes, lack of control groups, heterogeneous outcome measures, and short or inconsistent follow-up intervals. Based on GRADE criteria, the overall certainty of evidence supporting efficacy and safety of spine interventions in the pediatric population was ‘very low’ (Table 2).

**Table 2:** GRADE level of evidence

Outcomes	Limitations	Inconsistency/Heterogeneity	Indirectness	Imprecision	Publication bias	Mean difference (95% CI)	Number of participants (studies)	Quality or certainty of the evidence (GRADE)
Pain at follow-up, assessed with various pain outcomes	Inconsistent and no steps taken to provide adequate control groups	High study design, intervention, and outcome reporting heterogeneity, limiting statistical analysis	Potential indirectness*	Optimal information size not met	Not detected	Unable to assess due to heterogeneity	258 (8 studies)	very low
Adverse Events	Inadequate outcome definition and capture	High reporting heterogeneity, underreporting possible	Potential indirectness*	Not detected	Not detected	Unable to assess	2 (2 studies)**	very low

*Direct evidence consists of research that directly compares the interventions which we are interested in, delivered to the populations in which we are interested, and measures outcomes important to patients. Indirectness may be present due to variations in axial pain etiology, as well as differences in treatment choice, medication dosing, and procedural technique across studies

**One additional study reported that 1 patient required a repeat same-level procedure at 18 month follow-up due to symptom recurrence"

## Discussion

### Background

While conservative treatment modalities for pediatric spinal pain including education of the patient and family members, activity modifications, therapeutic rehabilitation, and targeted pharmacologics are strongly advised as initial modalities, there is a gap in the literature discussing the efficacy and safety of interventional modalities as a next line of treatment [24]. This gap is an incredibly important area of research warranting further attention. There are practice guidelines for other realms of pediatric pain management, for example the appropriateness of imaging for pediatric lower back pain, opioid prescription for acute pain in pediatric populations, as well as acute pain management in the perioperative setting [1, 25, 26]. However, there is a lack of practice guidelines regarding interventional spinal modalities targeting chronic neck and back pain for pediatric populations. There are likely many factors contributing to this gap, including hesitation from providers to offer spinal interventions to pediatric patients, concern for the potential long term side effects of interventions given limited long term follow up data, as well as a lack of formal guidelines.

### Overall Findings

This systematic review screened 490 studies, eight of which met inclusion criteria, and thus a total of 258 pediatric patients were included. To date, this analysis includes the largest subset of pediatric patients assessed who underwent minimally invasive interventions for neck and back pain. As there is a scarcity of literature discussing potential interventions in the management of pediatric spinal pain, this review fills a critical gap in the literature.

While all spinal pain pathologies were included in the search criteria, the existing literature predominantly examines lumbar based interventions. The most common conditions included nonspecific lower back pain, herniated discs with or without radiculopathy and sacroiliac joint pain. Other less common conditions were identified such as tethered cord syndrome, Baastrup's disease, and sacral facet fractures. A range of interventions were examined, including most commonly epidural steroid injections, in addition to medial branch blocks, sacroiliac joint injections, ozone discolysis, interspinous ligament injections, and microdiscectomies.

Subjective pain relief scales were the most commonly utilized outcome, though validated measures such as VAS and ODI were also utilized. Collectively, our evidence suggests that spinal interventions may be beneficial in the management of pediatric neck and back pain. Moreover, interventions may also be beneficial for functional outcomes, though significantly more research is needed to evaluate this. AEs were generally infrequent and transient if present. These findings are consistent with reviews examining rates of AEs in adult populations undergoing interventional pain procedures, one of which reported an overall complication rate of 1.9% across 26,061 procedures, with no major complications [27]. AEs reported in this review included a sciatic nerve block following a sacroiliac joint injection, in addition to a dural puncture following an epidural steroid injection requiring an epidural blood patch [16, 20]. Both are potential complications also noted in adult populations, with sciatic nerve blocks following sacroiliac joint injections reported infrequently across studies and dural punctures following epidural steroid injections occurring in < 0.01% of cases [27, 28]. While a more robust assessment is needed to further validate these findings, these findings suggest that interventional pain procedures for pediatric patients have a comparable safety profile to that of adult populations.

### Clinical Implications

There is a stark lack of literature examining the effectiveness and safety of interventions for the management of refractory neck and back pain. This systematic review provides an overview of nonoperative spinal interventions utilized in the management of pediatric neck and back pain. The authors of this review highly encourage exhaustion of conservative treatment modalities, including activity modifications, therapeutic rehabilitation, and pharmacologics, prior to consideration of interventional modalities. Additionally, appropriate patient selection remains key to treatment success. Without practice guidelines, the choice of intervention revolves around clinical judgement and joint decision making with the patient and family.

### Limitations of Studies

We acknowledge the potential limitations of this study. First, both the small number of studies and patients that were eligible for inclusion increases the potential impact of bias. This reflects the scarcity of literature examining interventions geared towards managing neck and back pain in pediatric populations. The lack of control groups in all the included studies severely limits the clinical impact and generalizability of the findings, as do the variations in prior treatment modalities. As such, the observed improvement may be due to a number of potential factors, including the natural progression of the condition, placebo effect, or other variables unable to be accounted for. No effect sizes were reported in any of the included studies, making it difficult to quantify the magnitude of impact these interventions had on spine pain. Moreover, a significant deal of heterogeneity exists in the underlying conditions, interventions employed, and outcome measures. Because of this, we were unable to complete a meaningful meta-analysis to offer increased certainty in the results of this study. Furthermore, over 80% of the patients included in this review underwent ESIs with limited representation from other types of spinal interventions, thereby limiting the generalizability of these findings.

## Conclusions

This review, shaped by the limited available literature and the heterogeneity of study populations, interventions, and methodologies, underscores the considerable variability in the management of pediatric neck and back pain. Though preliminary evidence suggests that the existing interventions may offer benefits in terms of pain relief and functional improvement for pediatric patients nonresponsive to conservative measures, future studies are needed to reduce confounding variables and better define the pediatric populations most likely to benefit from spinal interventions. Development of future practice guidelines would also help reduce variability across clinical practices and improve patient care.

## Key References

- Chambers CT, Dol J, Tutelman PR, et al. The prevalence of chronic pain in children and adolescents: a systematic review update and meta-analysis. *Pain*. Oct 1 2024;165(10):2215-2234. 10.1097/j.pain.0000000000003267
  - ○ This recent and comprehensive meta-analysis authoritatively establishes the high prevalence of chronic pain in children, finding that approximately 1 in 5 experience chronic pain. Citing this high-quality study underscores the clinical importance and scale of the problem, providing a strong rationale for our review of potential treatment modalities.
- Kurgansky KE, Rodriguez ST, Kralj MS, et al. Epidural Steroid Injections for Radiculopathy and/or Back Pain in Children and Adolescents: A Retrospective Cohort Study With a Prospective Follow-Up. *Reg Anesth Pain Med*. Jan-Feb 2016;41(1):86–92. 10.1097/aap.0000000000000338
  - ○ As the largest study included in this review, this retrospective cohort of 224 pediatric patients provides the most substantial evidence on the safety and outcomes of epidural steroid injections in this population to date. The study reported no serious adverse events and noted favorable long-term pain and function scores, forming the cornerstone of our analysis and heavily informing this review’s conclusions regarding the procedure’s safety profile in pediatric patients.
- Sayed D, Grider J, Strand N, et al. The American Society of Pain and Neuroscience (ASPN) Evidence-Based Clinical Guideline of Interventional Treatments for Low Back Pain. *J Pain Res*. 2022;15:3729-3832. 10.2147/jpr.S386879
  - ○ This extensive evidence-based guideline for interventional treatments in adults serves as a crucial benchmark, effectively highlighting the significant evidence gap and lack of formal guidance for the pediatric population. The comprehensive nature of these adult recommendations underscores the novelty and importance of this review, which aims to consolidate the sparse available literature for children and adolescents to inform future clinical practice.

## Supplementary Information

Below is the link to the electronic supplementary material.

Supplementary file1 (XLSX 521 KB)

## Data Availability

No datasets were generated or analysed during the current study.
